# Multi-Scale Imaging and Informatics Pipeline for In Situ Pluripotent Stem Cell Analysis

**DOI:** 10.1371/journal.pone.0116037

**Published:** 2014-12-31

**Authors:** Bryan R. Gorman, Junjie Lu, Anna Baccei, Nathan C. Lowry, Jeremy E. Purvis, Rami S. Mangoubi, Paul H. Lerou

**Affiliations:** 1 Department Of Pediatric Newborn Medicine and Department of Medicine, Division of Genetics, Brigham and Women's Hospital; Harvard Medical School; Harvard Stem Cell Institute, Boston, Massachusetts, United States of America; 2 Harvard-MIT Division Of Health Sciences and Technology, Massachusetts Institute Of Technology, Cambridge, Massachusetts, United States of America; 3 Charles Stark Draper Laboratory, Cambridge, Massachusetts, United States of America; 4 Department Of Genetics, University of North Carolina School of Medicine, Chapel Hill, North Carolina, United States of America; Hemocentro de Ribeirão Preto, HC-FMRP-USP., Brazil

## Abstract

Human pluripotent stem (hPS) cells are a potential source of cells for medical therapy and an ideal system to study fate decisions in early development. However, hPS cells cultured *in vitro* exhibit a high degree of heterogeneity, presenting an obstacle to clinical translation. hPS cells grow in spatially patterned colony structures, necessitating quantitative single-cell image analysis. We offer a tool for analyzing the spatial population context of hPS cells that integrates automated fluorescent microscopy with an analysis pipeline. It enables high-throughput detection of colonies at low resolution, with single-cellular and sub-cellular analysis at high resolutions, generating seamless *in situ* maps of single-cellular data organized by colony. We demonstrate the tool's utility by analyzing inter- and intra-colony heterogeneity of hPS cell cycle regulation and pluripotency marker expression. We measured the heterogeneity within individual colonies by analyzing cell cycle as a function of distance. Cells loosely associated with the outside of the colony are more likely to be in G1, reflecting a less pluripotent state, while cells within the first pluripotent layer are more likely to be in G2, possibly reflecting a G2/M block. Our multi-scale analysis tool groups colony regions into density classes, and cells belonging to those classes have distinct distributions of pluripotency markers and respond differently to DNA damage induction. Lastly, we demonstrate that our pipeline can robustly handle high-content, high-resolution single molecular mRNA FISH data by using novel image processing techniques. Overall, the imaging informatics pipeline presented offers a novel approach to the analysis of hPS cells that includes not only single cell features but also colony wide, and more generally, multi-scale spatial configuration.

## Introduction

Ever since human embryonic stem cells (hES) cells were first isolated from the inner cell mass of a human blastocyst [1], they have been viewed as a ‘holy grail’ of medical promise. Because they have the ability to self-renew indefinitely and differentiate into any cell type of the body, they are potentially an unlimited source of cells for patients in need of cellular therapy [2]. Moreover, due to their provenance, hES cells are an ideal system to study cellular fate decisions in early human development. More recently, Yamanaka and colleagues devised a method to convert fully differentiated somatic cells into an embryonic-like state, known as induced pluripotent stem (iPS) cells, through the over-expression of certain transcription factors [3], [4]. Collectively, we refer to hES cells and iPS cells as human pluripotent stem (hPS) cells.

**Figure 1:**
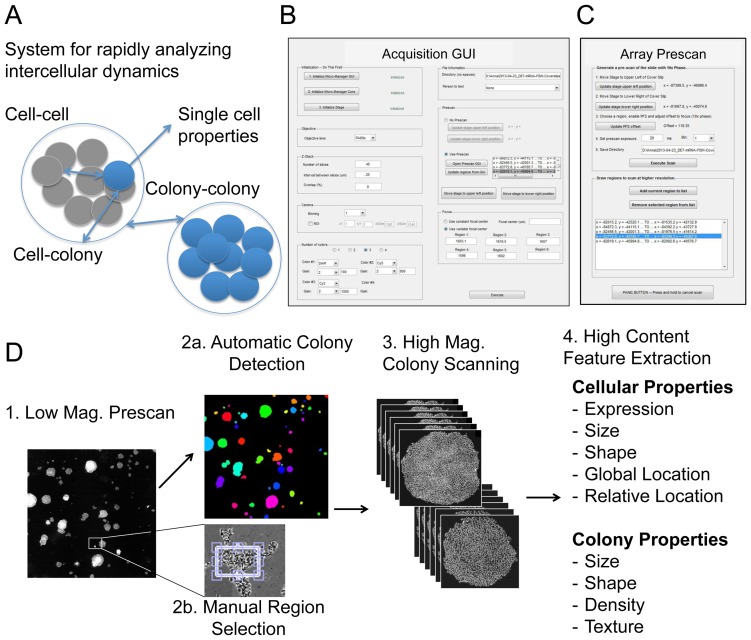
Overview of the multi-scale Imaging and Informatics pipeline. (A) Our system enables researchers to analyze intercellular dynamics in hES cells by structuring relationships between cells within a colony; between cells and the colony they belong to; and from one colony to another. (B) The window of the main GUI controlling the automated image acquisition software. (C) A daughter window of the GUI facilitating pre-scanning of the slide and selection of regions. (D) A workflow to obtain image-derived features from single cells, while placing them in the context of the colony they belong to. Thus, the pipeline involves a multi-scale segmentation of the colonies within the sample, and the cells within the colonies. Each cell not only has a physical address within the colony, but is linked with colony-wide properties, such as the size and shape of the colony it is derived from.

A major branch of therapeutic stem cell research is aimed at understanding how pluripotent cells acquire their ultimate fate as a defined tissue. Considerable effort has gone into developing directed differentiation protocols by empirically adding or removing inductive signals to the differentiating cell population in order to progressively enrich specific cell subsets that will yield the cell of interest [5], however current directed differentiation protocols are often low yield and highly variable. Compounding the complexity of *in vitro* differentiation is that hPS cells are inherently highly heterogeneous (Fig. 1A). Heterogeneity (cell-to-cell phenotypic variation) is a consistent and necessary feature of hES cells [6], [7]. Lineage-biased progenitor cells, identified by expression of specific cell-surface markers, can be isolated from a clonal population of undifferentiated hES cells [8]. This inherent heterogeneity is thought to contribute to the ability of hES cells to differentiate into multiple lineages [6]. Nevertheless, it poses problems for the clinical use of pluripotent stem cells by biasing subsets of cells to different lineages.

An additional source of heterogeneity is induced as an artifact of the cell culture micro-environment, and includes such features as proximity to other cells, density, and gradients in growth factors and other cytokines. hES cells share direct cell-to-cell contacts in the form of gap junctions [9]; are maintained through diffusible autocrine and paracrine signaling [10]; display high rates of apoptosis when plated as single cells [11]; and undergo anoikis [12]. The colony is a feature of standard hES culture conditions. Standard hES cultures exhibit a wide diversity of colony and cellular phenotypes. Presumably, cells in large, dense, colonies receive a different set of chemical and mechanical signals than cells residing in smaller, sparser, colonies. Moreover, within any given colony, there may emerge cellular subsets that spontaneously differentiate from hES cells and help to support the growing colony [13].

Population context has been shown to correlate with heterogeneous cellular states in other cell types [14]–[17], and we hypothesize that it is vital to understanding hPS cell heterogeneity. Studies have started to explore the structure of the hES cell niche and how ES cells self-organize into subpopulations, but a complete understanding of cellular dynamics within the colony structure remains elusive. Traditional molecular biology approaches such as immunoblotting or gene expression analysis average cells over the population, which can conceal the true cellular dynamics [18]. And, while flow cytometry provides single cell data, it requires breaking up colonies to create cellular suspensions.

Thus, novel imaging approaches are required. Recent studies have developed methods for analyzing stem cell colonies. We and others have reported image analysis pipelines for segmenting stem cell colonies and performing location analysis on specifically-labeled cells [19]–[24]. Alternatively, others have developed automated analysis and tracking systems to monitor the growth and morphology of live colonies over time, using phase contrast light [25], [26]. The method we describe builds upon and extends these works by integrating automated fluorescent microscopy with an analysis pipeline that enables high-throughput detection of colonies at low resolution, combined with single-cellular and sub-cellular analysis at high resolutions, generating seamless cell maps and single-cellular data organized by colony and neighborhood. Furthermore, it allows analysis of samples where the geometry and spatial configuration of the colonies is unconstrained. Additionally, our approach presented here differs from these methods for analyzing stem cell colonies in that it is, among others, multi-scale. For instance, it focuses on generating *in situ* maps of all cells and their locations relative to each other, as well as within the structure of the colony, allowing comparison across multiple length scales. This rigorous informatics approach will lay the foundation for understanding the influence of spatial population context in early differentiation.

## Methods

In this section, we describe the experimental methods used to generate the data samples that are later analyzed (Sections 1–2). Then, we describe main aspects of the tool itself: the automated imaging of the slide and high magnification imaging of colony regions (Section 3), the image processing of colony and cellular images (Section 4), and the construction of the data into seamless cellular maps that are consistent across adjacent fields (Section 5).

### 1 Cell culture

hESC (CHB-8 [27]) cells were maintained on irradiated CF-1 MEFs (GlobalStem) in DMEM/F12 supplemented with 20% KnockOut Serum Replacement (Gibco), 0.1 mM 2-mercaptoethanol (Gibco), 13 GlutaMAX (Gibco), non-essential amino acids (Gibco), Beta-mercaptoethanol, and 10 ng/ml FGF2 (Sigma), or in feeder free conditions on Matrigel in mTeSR or E8 medium with 5× supplement (Stem Cell Technologies). For imaging experiments, cells were typically plated on matrigel-coated glass bottom culture dishes (MatTek). Differentiation of hESCs was induced with Activin A [28] or retinoic acid. To label S phase cells, cultures were pulse labeled with EdU for 30 minutes.

### 2 Sample staining

For immunofluorescent staining, cells were stained according to Lu et al., 2014. Primary antibodies used were Oct4 (Abcam 19857), Nanog (Abcam 21624), phospho-H3 (Millipore 06-570), and cPARP (Epitomics 1074-1). Secondary antibodies were Alexa Fluor 594 anti-goat IgG or Alexa Fluor 488 anti-rabbit IgG (both from Life Technologies). EdU staining was performed according to manufacturer's instructions (Life Technologies). We found that placing the dish on a shaker resulted in the most even antibody distribution. For single molecule mRNA FISH (smFISH) hybridization, Custom Stellaris mRNA probes were obtained from Biosearch Technologies. Hybridization was performed following a published protocol [29].

### 3 Microscopy

We utilized a Nikon Eclipse Ti inverted microscope equipped with a Prior motorized stage and a high-resolution CoolSNAP HQ2 CCD camera. We wrote custom software in MATLAB to control the microscope, camera, shutters, and stage hardware. This software utilizes the Micro-Manager API [30], allowing it to be easily adapted to other hardware configurations.

We constructed a program that allows multi-scale imaging of ESCs in different regions across the slide. A detailed walkthrough of this software is provided in S1 Text. In order to facilitate usage by people with varying computer backgrounds, we also constructed a GUI to control this program. The main window of the GUI is shown in Fig. 1B. The main GUI window contains UI elements that allow the user to adjust all necessary parameters of the program. This includes parameters controlling the microscope nosepiece (objective lens to be used), the camera (binning, gain, exposure, and region of interest properties), the z-drive (number and interval of z-stack slices), and the program I/O (save directory, notification of completion).

The second major element of the program is region selection. In selecting regions, the user has the option of generating a lower-magnification pre-scan imaged with transmitted phase light and/or fluorescent channels. The pre-scan sub-GUI is illustrated in Fig. 1C. Once the pre-scan is executed, bounding boxes are automatically drawn around colonies through integrated processing and segmentation of the colonies from the pre-scan image. Alternatively, the user may manually select specific regions of interest by drawing boxes onto macroscopic image of the entire slide (Fig. 1D). Selected regions can then be flexibly reviewed and edited before the region positional data is transferred into the main program for execution of the final full-content image acquisition.

The software offers many flexible options for the high-resolution scan. Users may select any level of magnification, including both dry and oil-immersion objective lenses; any combination of fluorescent filters and exposure times; and may opt for an overlap between adjacent fields. Additionally, we implemented the ability to image Z-stacks, which may be useful for many applications such as detection of single molecule mRNA FISH probes (Fig. 4). Because the focus level may vary across a slide, users have the option of automatic focusing using the microscope hardware (e.g. Nikon Perfect Focus), or manually defining a constant or variable focal plane for each selected region. As the program scans the selected regions, tiff stacks are saved in a specified directory, along with data files recording the imaging parameters and each stage position. Finally, a composite array of each region is constructed, by tiling together maximum projections of each tiff stack.

This system provides powerful and flexible controls to image and analyze the heterogeneous population context of hPS cell culture at multiple scales, from molecular level, to cell, to colony regions, to colonies. Because it seamlessly integrates imaging with analysis, including automatic region selection based on colony segmentation, our setup is an improvement over currently available solutions to study long-range relationships of single hPS cells in culture.

### 4 Image analysis and informatics

#### 4a. Colony segmentation and identification

We mosaicked constituent field images into complete regions, taking advantage of the submicron accuracy of our Prior ProScan III stage. Using a combination of local neighborhood Gaussian blurring and thresholding using Otsu's method [31], we identified the boundaries of the colonies. Those colonies were then cropped from the mosaic and sent to the segmentation module. Due to slight discontinuities at the borders between regions, cells on the borders were discarded from the analysis.

#### 4b. Segmentation of cells and feature extraction

Field images were loaded into the segmentation module for processing and feature extraction. CellProfiler [32], [33] was called directly from MATLAB, with the resulting segmentation label matrices and feature sets automatically reimported. Within CellProfiler and MATLAB, we extracted numerous features from cells at one scale, and from colonies, at a higher scale. Features include integrated intensity of multiple stains, simple measures of texture, and shape measurements, such as area, perimeter, and eccentricity. Additional regional and global properties were calculated and appended to the data structure. These include regional and global indices for each cell, regional and global virtual addresses and physical addresses, and levels of DAPI and other relevant staining signals.

#### 4c. Microscopy alternative

The analysis and informatics pipeline is easily portable. As an example, images acquired with Laser Scanning Cytometry (CompuCyte; Cambridge, MA), a non-confocal laser scanning microscope, can also be processed; the tool is thus capable of multimodal data fusion, at least at the classification or decision level.

#### 4d. Data output

The software outputs either a nested MATLAB data structure or a CSV file. The data include the colony label and address of each cell, linked with the properties of those colonies (size, shape, etc.), allowing for intra-colony and inter-colony analysis.

#### 4e. Data visualization with FCS Express

We wrote custom scripts to export data into the FCS Express 4 Image Cytometry format (De Novo Software), allowing flexible manipulation of image and feature data.

### 5 Construction of a virtual slide

The virtual slide abstracts the important information about cellular space without having to deal with massive, cumbersome image files; an attribute particularly important when data are multimodal. The segmentation label matrix for each field is loaded into the region label matrix. Field labels are converted to global labels. For each field, the feature data are loaded and collated in to a data array structure addressed by the region number, field coordinates, and feature of interest. The centroid of each cell is derived from the segmentation results. Those values were combined with the colony identification step to yield an “address” of the cell within the context of its neighbors and the colony in which it resides.

At a higher scale, our system incorporates a method for generating seamless maps of cellular fields (S1 Fig.). We apply a heuristic based on the accuracy of our robotic stage, which is that the same object existing in adjacent fields should overlap in pixel space. As long as the overlapped margin is at least twice as large as the diameter of a large cell, all cells will completely exist in at least one field (S1 Fig.). Corresponding cellular objects are identified across image fields, and a consensus seamless segmentation is arrived at by taking the larger of the two objects.

## Results

We analyzed quantitative relationships of individual cell properties to their location within the colony and colony-wide features such as size, shape, and texture. These properties include cell cycle state, expression of pluripotency markers such as Oct4 and Nanog, and shape and size of the individual nuclei. In addition, we used this tool to examine relationship of cell cycle variation as a function of colony properties and cellular position.

### Cell cycle heterogeneity as a function of location within colonies

Cell cycle regulation and pluripotency are closely linked; changes in pluripotency are coupled to changes in cell cycle progression [34]. We used our platform to investigate whether cell cycle varies as a function of cellular location within a colony. 80 CHB-8 hES colonies across two coverslips were imaged for DAPI, EdU (S phase), phospho-H3S10 (M phase), and the pluripotency marker Nanog. Taking the processed data, we generated a scattered density plot of single-cell values of mean intensity of the EdU stain versus integrated intensity of DAPI and categorized cells into G1, S, and G2/M subpopulations corresponding to their progression through the cell cycle (S2A Fig.). The mean Nanog intensity distribution of these subpopulations showed that the G1 subpopulation was enriched for cells with low Nanog levels. Approximately 10% of G1 cells were Nanog negative, versus just 1% of S and G2/M cells (S2B Fig.). This finding is consistent with the observation that pluripotent cells transition through G1 phase quickly, and transition more slowly as they differentiate [35].

**Figure 2:**
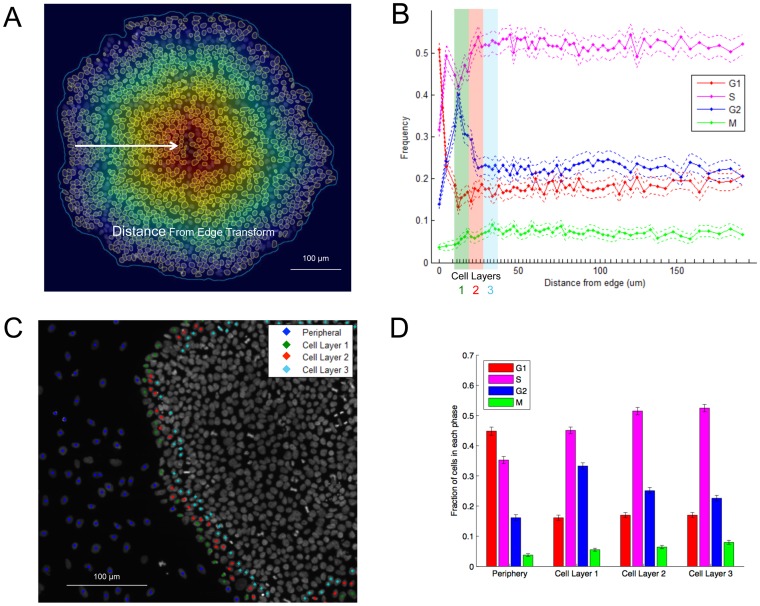
Spatial enrichment of G1 and G2 phase cells in colonies. (A) Illustration of the distance transform applied to cells in all colonies. Outside of the boundaries of the colony, the distance from edge is “0”, and within the border of the colony, the distance ranges between 0 and the maximum radius of the colony. (B) Frequencies of cells as a function of distance from edge for the G1 (red), S (magenta), and G2 (blue) and M (green) subpopulations. Error bars indicate the 95% confidence interval based on 1000 bootstrap samples. Abscissa tick marks indicate the distance intervals from which the population and bootstrap frequencies were calculated. Each distance interval contains approximately 1500 cells. The data points are located halfway in between each interval. The frequencies at each point add up to 1. Of note is the statistically significant peak in the G2/M subpopulation at 25 microns. (C) A cellular map illustrating the periphery (blue), Cell Layer 1 (green), Cell Layer 2 (red), and Cell Layer 3 (blue) subpopulations. (D) Distributions of cell cycle phases for each cell layer across many colonies. There is a significant enrichment of G2 cells in Cell Layer 1 relative to Cell Layers 2 and 3.

One of the features derived from our informatics pipeline is the distance of a cell from the edge of the colony it resides in. Using the distance transform illustrated in Fig. 2A, cells outside or peripheral to the colony have a distance of 0 from the edge of the colony, and the measurement increases from 0 to a maximum value equal to the shortest radial distance from the center of the colony to the edge. First, we partitioned the data set into intervals along the distance-from-edge axis with approximately equal number of cells in each bin. Logical gates were specified for cells in the G1, S, G2, and M subpopulations, as well as for each distance interval. The frequency of the G1, S, G2, and M subpopulations belonging to each distance interval was calculated. For each distance interval, we then resampled the data (with replacement) from the population 1000 times, and calculated the G1, S, and G2/M frequencies at that interval. The resampled frequencies had a normal distribution whose mean was approximately equal to the population frequency. Confidence intervals were calculated from 95% of the bootstrap samples.

The multiple scale capabilities helped uncover a noteworthy pattern (Fig. 2B): the frequency of G1, S, G2, and M subpopulations was largely stable over the span of cellular distances, except at the periphery of the colony (distance of 0), where G1 cells were enriched; and within the first cell layer from the edge (“Cell Layer 1,” i.e., the outermost ring of cells belonging to the colony), where G2/M cells were enriched. We then gated on the peripheral population with the G1 peak (Gate Periphery), and the populations within a distance of 1–3 cell diameters from the edge (Cell Layers 1–3). Fig. 2C showed that these gates correctly classified cells into the desired subpopulations. The peripheral subpopulation displayed a substantially enriched G1 peak (Fig. 2D) as well as substantially enriched Nanog negative levels of nearly 63% (S2C and S2D Figs.). In Cell Layer 1, we found a significant enrichment of the G2 population compared to Cell Layers 2–3. However, M phase cells were not enriched in Cell Layer 1, possibly suggesting a G2/M block. Nearly 100% of cells were Nanog positive in Cell Layers 1–3.

To check whether this was primarily a phenomenon of cells belong to colonies of a certain size, we divided cells into groups on the basis of they belonged to a small, medium, or large-sized colony. We then similarly assessed the cell cycle percentages of those different colony types in each of the identified Cell Layers. We found that cells of each colony size were enriched in S-phase in Cell Layer 1 relative to Cell Layers 2 and 3, though the enrichment was slightly stronger in small colonies (S3 Fig.), probably a result of rapid proliferation in particular for small colonies. The enrichment of G2 phase cells in cell layer 1 is similarly present in all small, medium, and large colonies. This analysis demonstrated the utility of the system in analyzing inter-colony heterogeneity.

Further understanding of the biological meaning of the enrichment of G2 phase cells at colony edge would require use of other methodologies, and measurement across different hES cell lines and culture conditions. However, it is a useful demonstration of the applicability of the tool we have described herein: novel insights on cell cycle progression by cell cycle determination of individual cells and analyzing their distribution relative to colony space across multiple colonies. Cellular decision-making is inherently stochastic and influenced by the cellular micro-environment, necessitating a high-throughput system capable of measuring and abstracting those relationships.

### Regional heterogeneity in cellular response to DNA damage

We previously reported that cellular differentiation state has a profound impact on DNA damage regulation [36]. Undifferentiated cells are highly sensitive to DNA damage, and will undergo apoptosis more readily. Differentiated cells, in contrast, are less sensitive to DNA damage. We hypothesized that colony-level heterogeneity may play a role in determining cell fate in response to DNA damage induction. To test this, we differentiated cells with retinoic acid (RA) for 0, 2 or 4 days. As expected, cells treated with RA exhibited the anticipated reduction in Oct4 (a pluripotency regulatory factor) and increased proportion of cells in G1 phase (S4 Fig.). After RA differentiation, wells were treated with neocarzinostatin (NCS), a radiomimetic drug that induces DNA double-strand breaks, for 0, 1, or 2 hours, and stained with cPARP antibodies to detect cells' apoptotic response.

**Figure 3:**
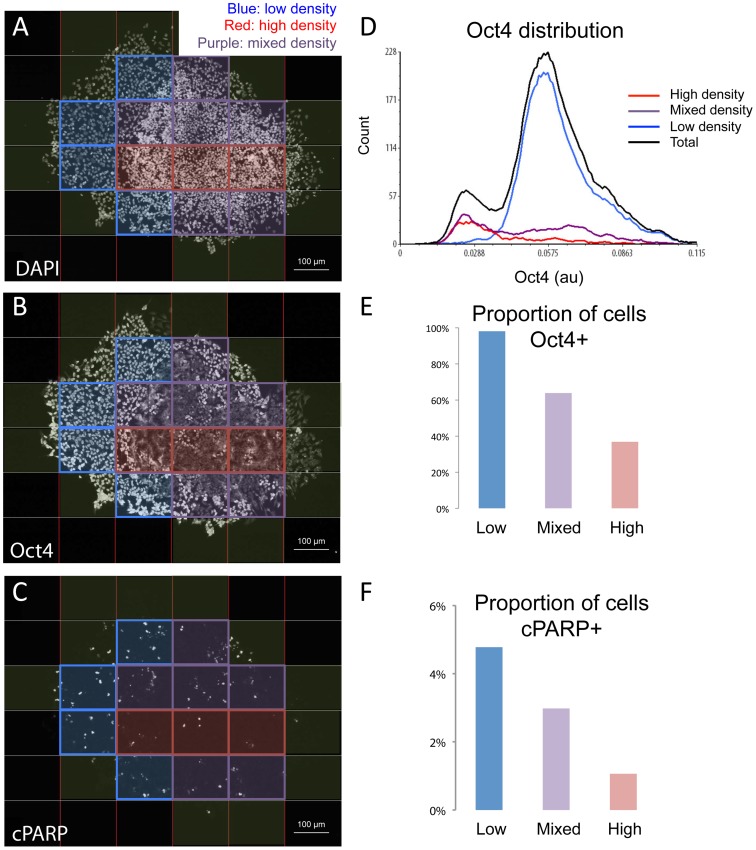
Cells in different regions of colonies respond differently to DNA damage. (A–C) In order to investigate regional heterogeneity, colonies of differentiated and NCS-treated cells were computationally divided into windows, which were then classified according to the density of cells contained. 25 colonies were divided into 164 windows, containing a total of 13,133 cells. As demonstrated, high cell density regions (purple) tend to have low Oct4 (B), and reduced induction of cPARP (C) upon DNA damage, than low (blue) and mixed (red) density regions. (D) Oct4 distribution of the cells in the above classified subregions. Black line shows the distribution of all cells. (E) Proportion of Oct4 positive cells in each subpopulation. (F) proportion of cPARP positive cells.

Looking at a higher level scale, the colony-level behavior of the cells within a single condition (after 1 day of RA differentiation), we found that instead of cells in all colonies responding in a uniform fashion to the external signals, there were clear regional behaviors from one colony to another and sometimes different patches within the same colony. Fig. 3A–3C is an example of one such colony we observed. This colony was a mosaic of undifferentiated and differentiated patches. Moreover, the undifferentiated patches were positive for Oct4 and more sensitive to DNA damage (had greater expression of cPARP), while the differentiated patches were negative for Oct4 and less sensitive to DNA damage (lower cPARP). In addition, we observed that the differentiated patches generally had a higher cellular density (possibly early stages of embryoid body formation), suggesting that gating cells according to cell density might reveal regional differences in expression of cPARP.

The cell density classifier from our image analysis tool did a reasonable job at separating cells into subpopulations. The high-density cell regions had a lower Oct4 distribution than the low-density cell regions (Fig. 3D), and the intermediate, or mixed subpopulation had Oct4 enrichment in between the two. Furthermore, the cPARP distribution was plotted and the cPARP positive subpopulation was gated (Fig. 3E). As expected, the lower density (undifferentiated subpopulations) had a greater proportion of cPARP+ cells (5%) than the mixed (2%) and higher density subpopulation (1%) (Fig. 3F). Moreover, using p53 as a positive control, we found that our measurement of staining intensity was not affected by cell density (S5 Fig.). Taken together, these results again underscore the utility of our tool to employ regional analysis of single-cell measurements that can reveal new physiological relationships.

### High Resolution Quantitative Analysis of Single molecule mRNA FISH (smFISH)

**Figure 4:**
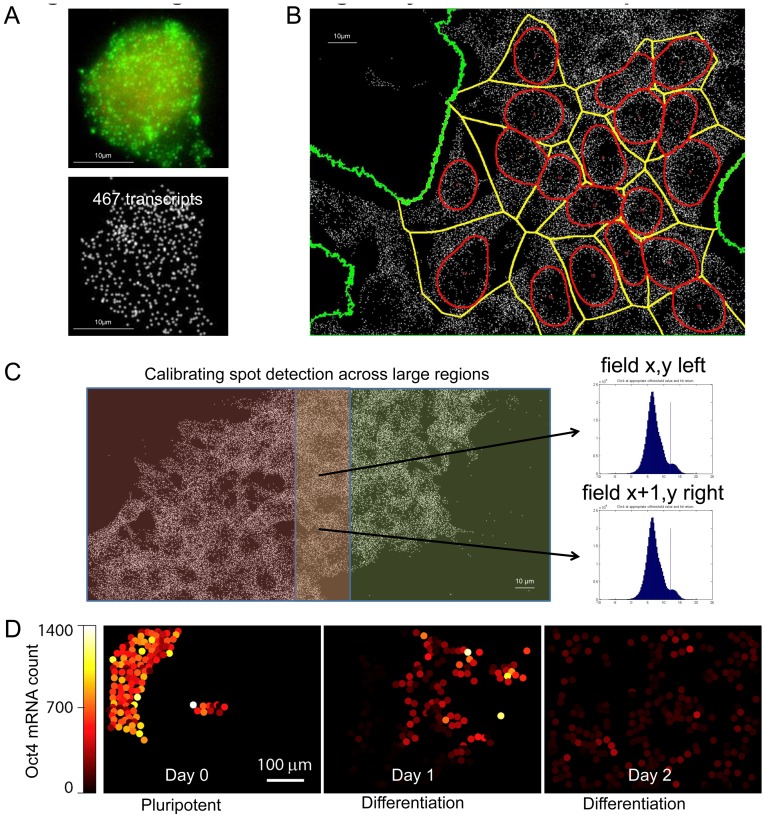
Large-scale mapping of single-cell heterogeneity in Oct4 mRNA expression. (A) Quantification of Oct4 mRNA level with smFISH in a hES cell. Top: raw image; Bottom: segmented FISH spots. (B) Segmentation of Oct4 spots in hES cells with nuclear boundaries in red, cell boundaries in yellow, and colonies boundaries in green. (**C**) Method for setting smFISH spot detection thresholds to ensure spot detection consistency across adjacent fields in a large region. (D) Reconstruction of Oct4 mRNA levels across 6 contiguous fields of view using the spot matching procedure detailed in the main text. Each circle represents an individual cell and circle color denotes the expression level of Oct4. Overall, Oct4 levels diminish through differentiation, although small clusters of cells with elevated Oct4 levels can be observed after 2 days of differentiation.

To test if the platform is amenable to datasets at the smallest scale of light microscope, we applied the imaging capture and analysis pipeline to single-molecule mRNA FISH (smFISH) data where each FISH spot corresponds to the diffraction-limited image of a RNA molecule with a size less than the resolution of the light microscope. To detect RNA spots computationally, we developed an algorithm that uses a morphological opening filter to remove out-of-focus light, followed by a Laplacian of Gaussian filter to sharpen the image and detect candidate foci [29]. A test statistic was derived for each foci based on the three-dimensional curvature of the spot (how much it resembles a point source of light) and the intensity of the spot [29]. This statistic is then thresholded to separate real foci from random noise. Additionally, a colony mask was generated using Otsu's method to threshold the maximum projection of the RNA background staining [31]. The outlines of segmented nuclei were expanded until touching, and the points at which they touched each other or the edges of the colony defined approximate cellular boundaries. Fig. 4A and 4B present examples of processed images of hES cells hybridized for Oct4 mRNA.

However, due to heterogeneity from one field to another, as well as potential diminution of the signal from adjacent fields due to imaging wide area, we found that it was difficult to ensure consistent spot detection from field to field. Therefore, we developed a method for adaptively adjusting the spot thresholds 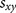 at each grid *x, y,* using adjacent overlapped fields (Fig. 4C). We subdivided each image space into top, bottom, left, and right margins. We then minimized an error function based on the sum squared differences in the number of detected spots at the overlapping margins between fields,
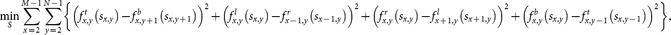
where *M* and *N* are the size of the regions in number of image fields and *f_x,y_* is the number of spots in the (*x,y*) image field calculated in the top (*t*), bottom (*b*), left (*l*), or right (*r*) margins using the threshold level *s_x,y_*. Minimizing the error function across all grids by locally or adaptively adjusting thresholds provides continuity and consistency as we move from one grid to another. The objective function uses a 2-norm, but a 1-norm may also be used for greater robustness to outliers. The minimization provided a matrix of local or grid specific threshold values *S = *[*s_x,y_*]. An initial estimate for *S, S_0_,* was obtained by thresholding the combined data for all fields and setting as constant. With this equalized spot detection, we are able to quantitate the FISH spots in each cell with high confidence, and follow the dynamic down regulation of Oct4 levels during differentiation of hES cells (Fig. 4D). This approach, which allows tiling of individual mRNA signals over multiple fields of view, allows large-scale analysis of heterogeneity in Oct4 levels of individual stem cells.

## Discussion

We present a multiple scale bioimage informatics platform designed to rapidly assay single pluripotent stem cell behavior in the context of subnuclear, cell and colony level environments. The platform proved to be user friendly; other individuals in our lab with varying computer backgrounds were able to use the software successfully. The platform enables integrated multiple scale imaging and analysis of pluripotent stem cell colonies, their constituent cells, nuclei, molecules and structures through manual or automated means. An integrated and portable data pipeline measures single-cell properties and places them within a hierarchical data structure corresponding to different scales of cellular micro-environment, in particular their spatial properties. Unlike most available commercial systems, where images are first stitched into a single large image before processed – drastically increasing the computational and memory requirements – our system creates consensus segmentation label matrices at the analysis stage, after applying segmentation algorithms to the individual fields. Moreover, it is easily extensible to diverse hardware configurations and imaging applications, such as volumetric analysis of cells and optical sectioning of 3D multicellular structures, particularly within the three-dimensional structures formed during stem cell differentiation.

Subsequently, we demonstrated the utility of this platform to analyze both inter- and intra-colony heterogeneity of human pluripotent stem cells. We found a link between cellular location within the colony and cell cycle regulation and pluripotency marker expression, which potentially signifies a novel finding that cells on the outer edge of the colony have slightly greater mitotic activity and contribute relatively more to colony growth. Additionally, we looked at regional heterogeneity of colonies varying by local cell density. When we separated subpopulations of cells belonging to different regional density classes, we found a stronger correlation between differentiation and DNA damage response than we had observed by simply aggregating all cells in the population. Discovery of these relationships would not be possible without multiple scale in situ imaging and analysis of many colonies simultaneously. With the ability to precisely quantify single-cell gene expression across many colonies, this approach may be used to discover subtle relationships between cells that explain the observed heterogeneity. For example, non-cell autonomous signaling between cells may generate certain expression patterns and spatial configurations within and between colonies. A better understanding of these factors will help guide perturbation strategies to control directed differentiation of pluripotent cells.

## Conclusions

We have created a tool for advanced tissue analysis that enables one to accurately measure cellular relationships over multiple length scales and resolutions of tissue morphology. In situ approaches are needed because they do not destroy the non-cell autonomous context of individual cells. This tool could be further applied to analyze other types of heterogeneous tissue, e.g. cancer cell aggregates. Further applications include analysis of heterogeneity at the transcriptional level and protein level during cell fate changes, and using data collected from live-cell imaging prior to fixation – thus establishing a framework for generation of high-quality data for modeling cellular decision-making in heterogeneous tissues.

## Supporting Information

S1 Fig **Construction of virtual slide.** Heuristic method for obtaining seamless segmentation fields using adjacent overlapping regions without stitching the raw image data directly.
(TIF)

S2 Fig **Cell cycle and Nanog distribution in hES colonies.** (**A**) Density scatter plot of integrated DAPI intensity versus mean EdU intensity, generated using FCS Express. Three gates were chosen to categorize cells based on their cell cycle state: G1, S, and G2/M. (**B**) Normalized histograms of mean Nanog intensity for the G1 (red), S (magenta), and G2/M (blue) subpopulations. (**C**) Differential spatial distribution of Nanog positive and negative cells in hES colonies (**D**). Histogram distribution of mean Nanog intensity of cells on periphery (green), Cell Layer 1 (yellow), Cell Layer 2 (cyan), and Cell Layer 3 (black) of the colonies.
(TIF)

S3 Fig **Inter-colony heterogeneity of cell cycle distribution.** (**A**) Histogram of cellular distance from edge in cells belonging to differently-sized colonies. The maximum cellular distance from the edge of the colony to the center was used to separate colonies into small (<150 um), medium (150–300 um) and large (>300 um) sizes. (**B**) Distributions of cell cycle phases for each cell layer in small, medium, and large-sized colonies. The inter-colony heterogeneity is not significant except for a slight enrichment in S-phase cells in Cell Layer 1 of small colonies. Error bars represent 95% of bootstrap samples.
(TIF)

S4 Fig **Phenotypic changes of hES cells during RA induced differentiation.** Cells undergoing RA-induced differentiation were stained for Oct4 and DAPI and analyzed with the pipeline. (**A**) Oct4 level goes down; and (**B**) more cells are in G1-phase as cells differentiate.
(TIF)

S5 Fig **Assessment of segmentation consistency and staining variation.** (**A**) Exemplary image of colony with 1 day RA differentiation and no NCS exposure. Entire sample contained 24,629 cells in 15 colonies divided into 410 sub-colony windows. Windows were 250 µm (width) by 192 µm (height) in size. (**B**) Integrated DAPI intensity over regional windows versus the number of segmented nuclei within the window. The relationship is linear over most densities, but is less linear at high densities where segmenting individual cells is more difficult and poorly segmented nuclei are discarded. Trend line is binned average +/− standard deviation. (**C**) Number of segmented nuclei within a window versus the average nuclear p53 content in that window. With no NCS treatment, nuclear p53 levels do not change as a function of cell density. The relationship is constant over the range of most densities. (**D**) In contrast to p53, nuclear Oct4 content decreases as a function of cell density.
(TIF)

S1 Text **Detailed Description of Program Operation.** Description of the source code availability, motivation, detailed image acquisition and analysis steps with 5 supporting figures (Figure S6–S10).
(DOCX)
