# Supplementary figures and images for: Multi-Scale Imaging and Informatics Pipeline for In Situ Pluripotent Stem Cell Analysis
Source: PLoS One. 2014 Dec 31;9(12):e116037. doi: 10.1371/journal.pone.0116037 (PMC4281228; doi:10.1371/journal.pone.0116037)

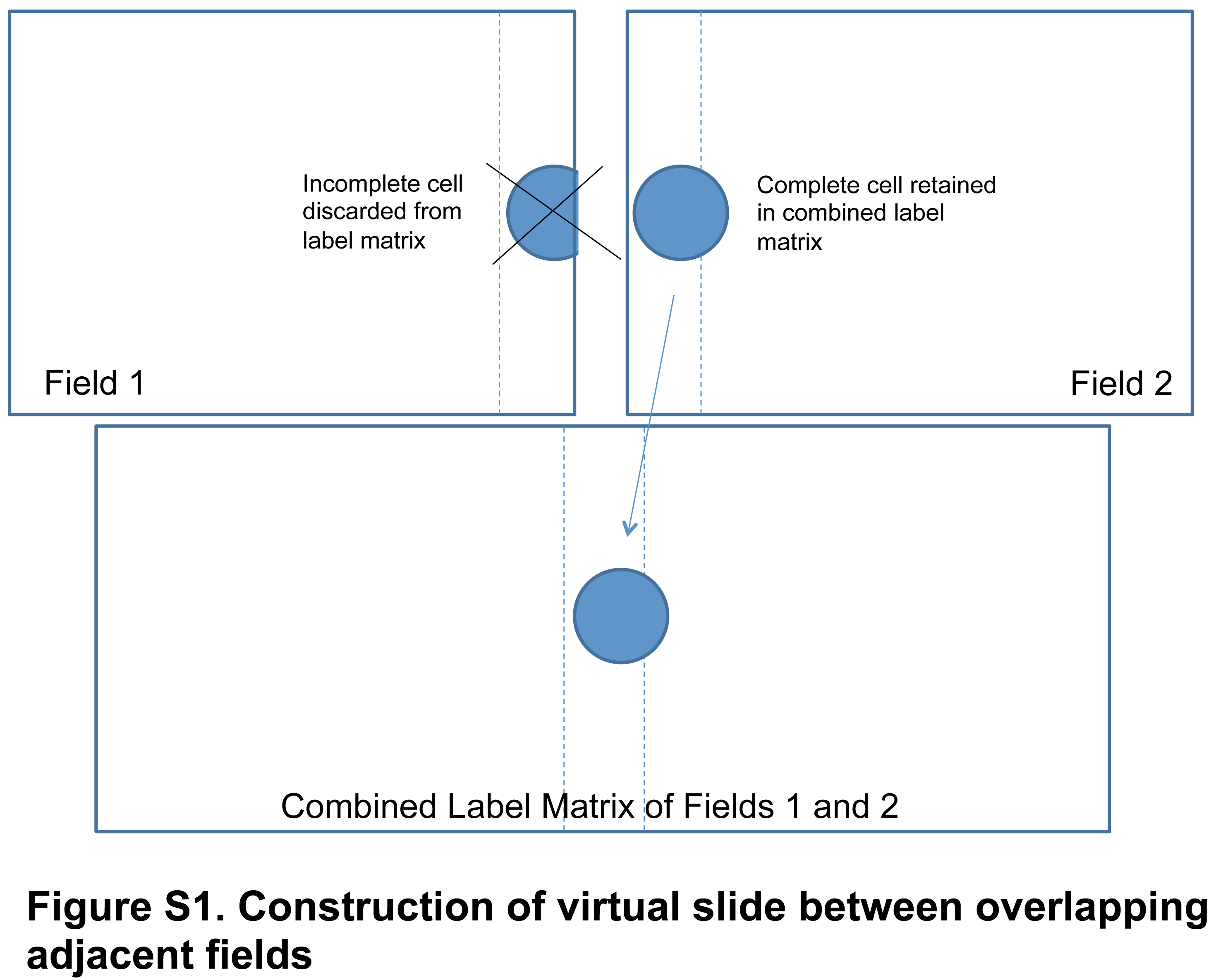

Supplement: S1 Fig — Construction of virtual slide. Heuristic method for obtaining seamless segmentation fields using adjacent overlapping regions without stitching the raw image data directly. (TIF) [file pone.0116037.s001.tif]

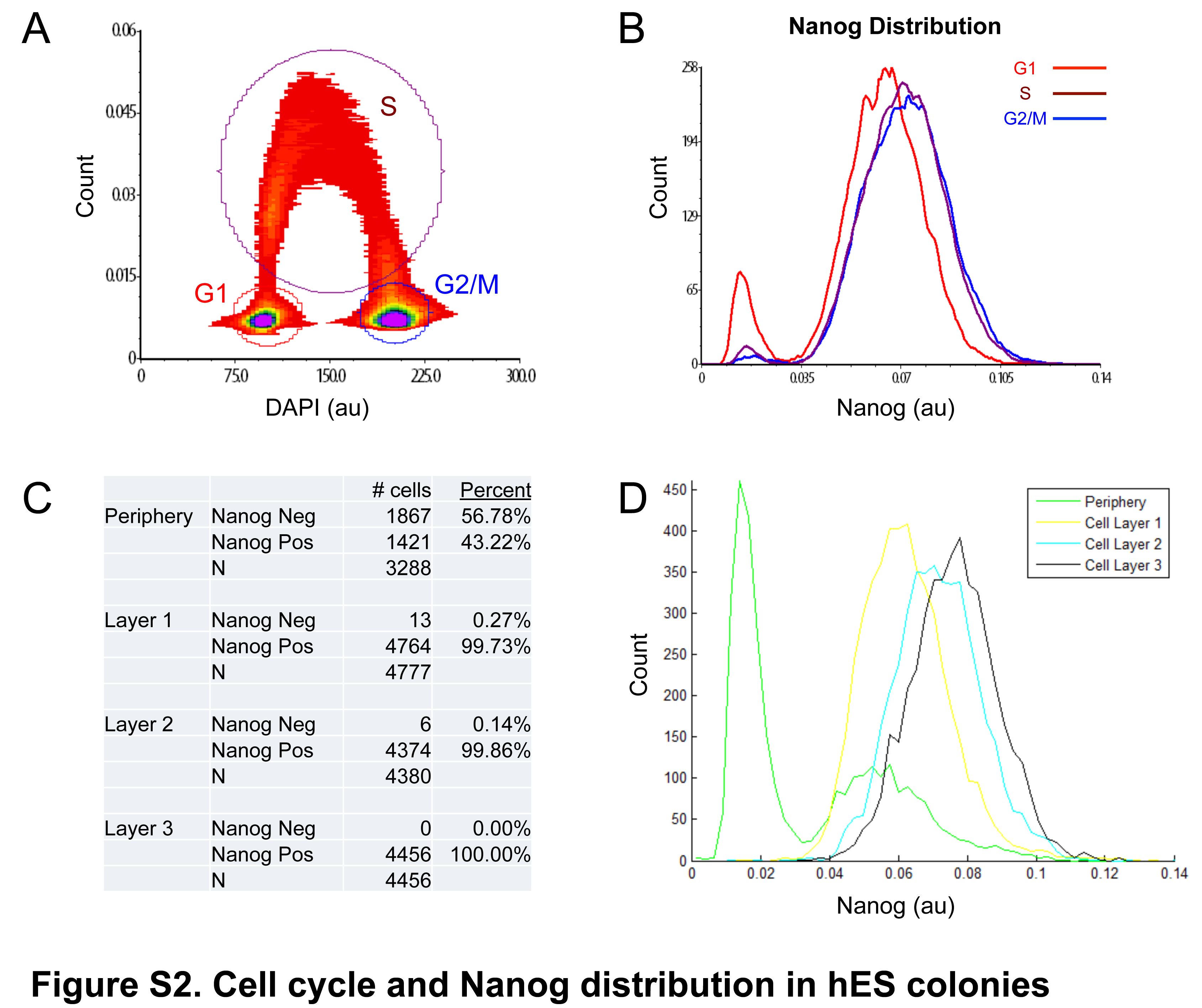

Supplement: S2 Fig — Cell cycle and Nanog distribution in hES colonies. (A) Density scatter plot of integrated DAPI intensity versus mean EdU intensity, generated using FCS Express. Three gates were chosen to categorize cells based on their cell cycle state: G1, S, and G2/M. (B) Normalized histograms of mean Nanog intensity for the G1 (red), S (magenta), and G2/M (blue) subpopulations. (C) Differential spatial distribution of Nanog positive and negative cells in hES colonies (D). Histogram distribution of mean Nanog intensity of cells on periphery (green), Cell Layer 1 (yellow), Cell Layer 2 (cyan), and Cell Layer 3 (black) of the colonies. (TIF) [file pone.0116037.s002.tif]

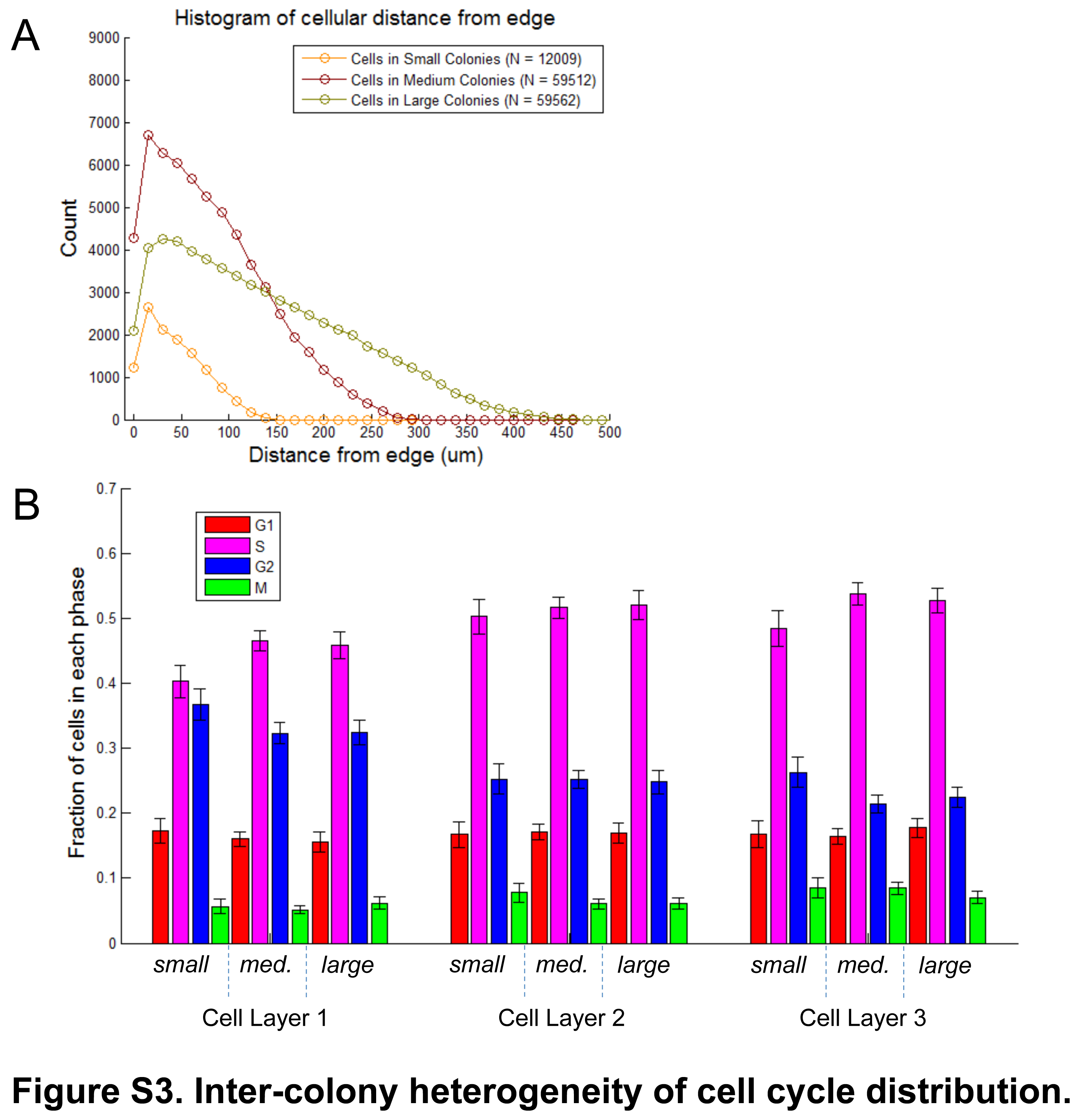

Supplement: S3 Fig — Inter-colony heterogeneity of cell cycle distribution. (A) Histogram of cellular distance from edge in cells belonging to differently-sized colonies. The maximum cellular distance from the edge of the colony to the center was used to separate colonies into small (<150 um), medium (150–300 um) and large (>300 um) sizes. (B) Distributions of cell cycle phases for each cell layer in small, medium, and large-sized colonies. The inter-colony heterogeneity is not significant except for a slight enrichment in S-phase cells in Cell Layer 1 of small colonies. Error bars represent 95% of bootstrap samples. (TIF) [file pone.0116037.s003.tif]

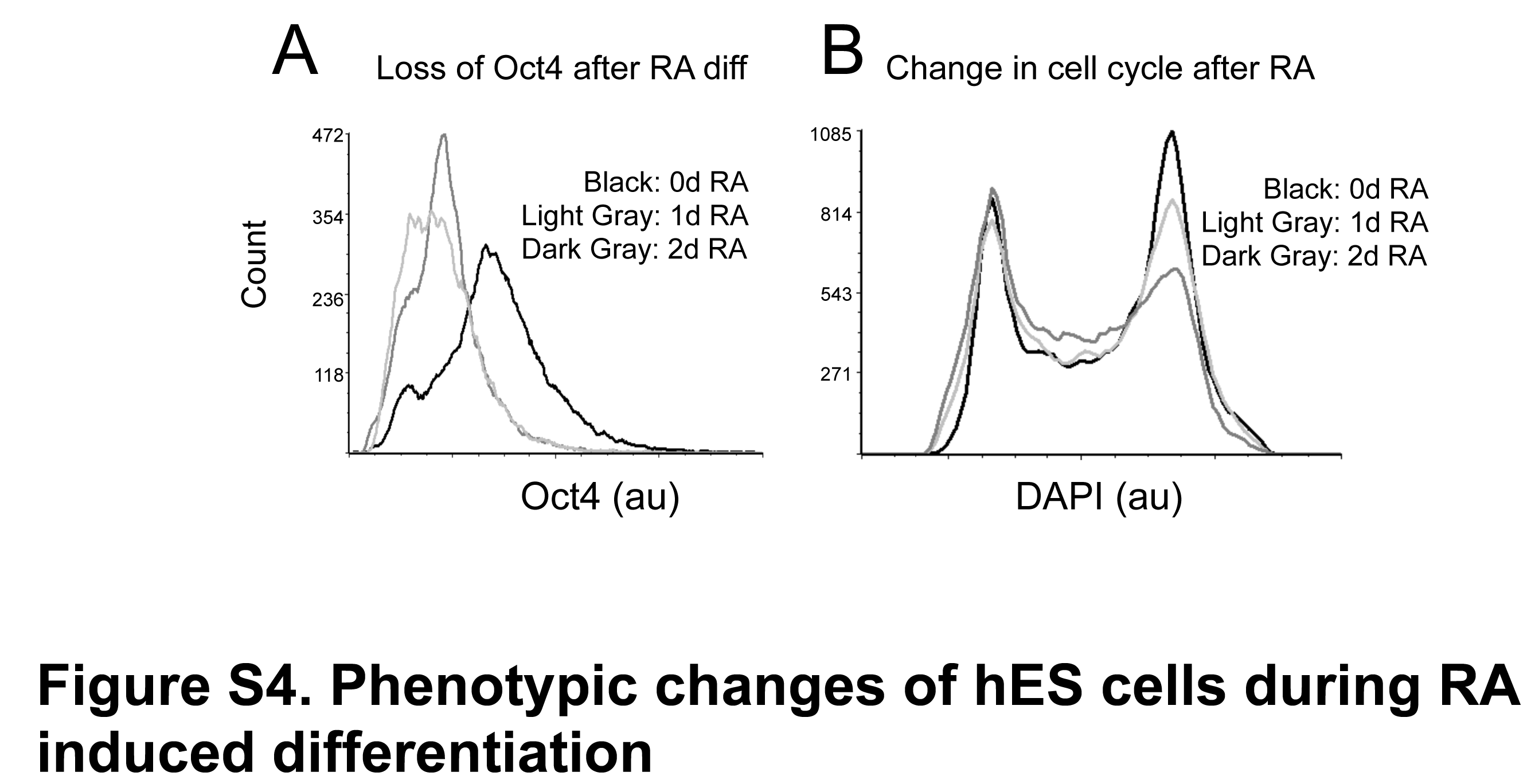

Supplement: S4 Fig — Phenotypic changes of hES cells during RA induced differentiation. Cells undergoing RA-induced differentiation were stained for Oct4 and DAPI and analyzed with the pipeline. (A) Oct4 level goes down; and (B) more cells are in G1-phase as cells differentiate. (TIF) [file pone.0116037.s004.tif]

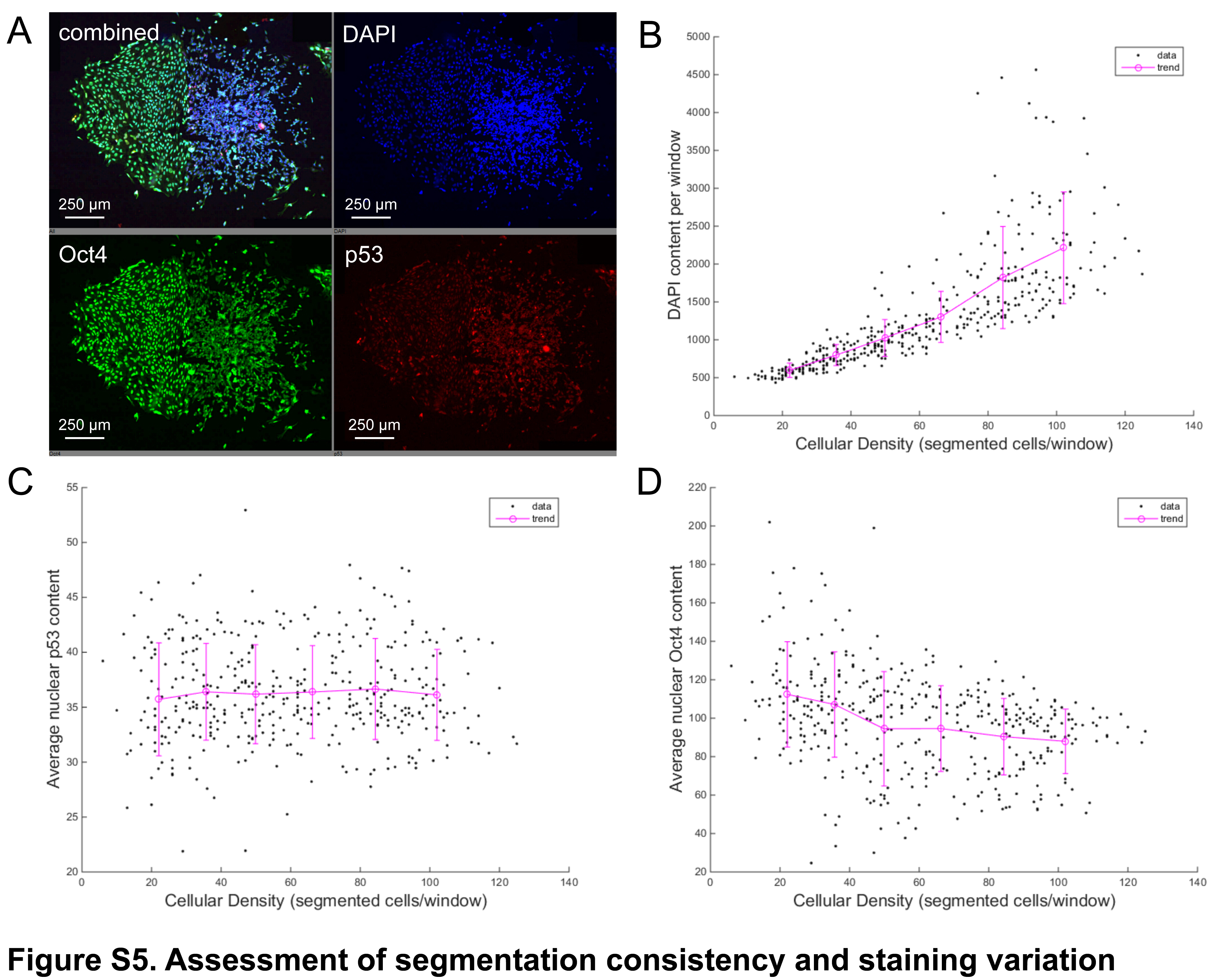

Supplement: S5 Fig — Assessment of segmentation consistency and staining variation. (A) Exemplary image of colony with 1 day RA differentiation and no NCS exposure. Entire sample contained 24,629 cells in 15 colonies divided into 410 sub-colony windows. Windows were 250 µm (width) by 192 µm (height) in size. (B) Integrated DAPI intensity over regional windows versus the number of segmented nuclei within the window. The relationship is linear over most densities, but is less linear at high densities where segmenting individual cells is more difficult and poorly segmented nuclei are discarded. Trend line is binned average +/− standard deviation. (C) Number of segmented nuclei within a window versus the average nuclear p53 content in that window. With no NCS treatment, nuclear p53 levels do not change as a function of cell density. The relationship is constant over the range of most densities. (D) In contrast to p53, nuclear Oct4 content decreases as a function of cell density. (TIF) [file pone.0116037.s005.tif]
