# Supplementary material for: Multi-Scale Imaging and Informatics Pipeline for In Situ Pluripotent Stem Cell Analysis
Source: PLoS One. 2014 Dec 31;9(12):e116037. doi: 10.1371/journal.pone.0116037 (PMC4281228; doi:10.1371/journal.pone.0116037)
Supplement: S1 Text — Detailed Description of Program Operation. Description of the source code availability, motivation, detailed image acquisition and analysis steps with 5 supporting figures (Figure S6–S10). (DOCX) [file pone.0116037.s006.docx]

Text S1:

Detailed Description of Program Operation

**AVAILABILITY**

Please visit <https://github.com/bgorm/lerou-stemcell> for updated information on usage and access to source code.

**MOTIVATION**

Human embryonic stem cells (hESCs) exhibit heterogeneity on multiple scales. Many high-magnification imaging applications such as single molecule mRNA FISH (smFISH) of hESCs are sufficiently time-consuming and labor-intensive that it may be difficult to capture enough data to get a sufficiently clear picture of the macro-scale heterogeneity that exists in the cell culture. To address this problem, we built a software program that allows high-magnification imaging within the context of the heterogeneity existing on the dish with smFISH as a working example.

**METHODS**

We utilized a Nikon Ti 2000 microscope, with a Prior ProScan III motorized stage, and a CoolSNAP-HQ2 high resolution CCD camera. Custom scripts were written in MATLAB to control the control the microscope, camera, and stage hardware. The Micro-Manager API ([valelab.ucsf.edu/~MM/MMwiki/](http://valelab.ucsf.edu/~MM/MMwiki/)) was used to control the microscope and camera drivers. The stage was independently controlled with commands issued through the serial port.

We constructed a program that allows multi-resolution imaging of embryonic stem cells in different regions across the slide. In order to facilitate usage by people with varying computer backgrounds, we also constructed a GUI to control this program. The main window of the GUI is shown in Figure S6. The main GUI window contains UI elements that allow the user to adjust all necessary parameters of the program. This includes parameters controlling the microscope nosepiece (objective lens to be used), the camera (binning, gain, exposure, and region of interest properties), the z-drive (number and interval of z-stack slices), and the program I/O (save directory, notification of completion).

The second major element of the program is region selection. In selecting regions, the user has the option of utilizing a lower-magnification prescan imaged with phase light. The prescan sub- GUI is illustrated in Figure S7. To generate a prescan, the program firsts asks the user to define the upper-left and lower-right positions of the slide, and then asks the user to enable perfect focus and set it to the correct offset to maintain proper focus, and finally the exposure level. Once the scan is executed, the user is presented with a macroscopic field of view of the entire slide, as shown in Figure S8a, which the user can then draw rectangles on to select regions. Alternatively, if fluorescent imaging is used, the rectangles outlining colony areas can be drawn automatically using an integrated segmentation algorithm. In order to optimize this to a particular sample, the user adjusts the four parameters shown Figure S9: hsize and sigma (properties of the Gaussian filter), threshold correction factor (a number that is multiplied by the Otsu threshold, such that >1 is a higher threshold and <1 is a lower threshold), and the minimum colony size (chosen such that randomly dispersed cells or debris are discarded).

Once the user has selected a region by drawing a rectangle around it, the user then adds the selection to a list. Users may see and edit previously selected region by highlighting selections in the textbox. Upon selecting all regions of interest, the user clicks an update button on the main GUI window to transfer the data into the main program for execution. Finally, because the focus level may change across a slide, users have the option of either using a constant focal center, or defining one for each region they select.

As the program is scanning the selected regions, tiff stacks are saved in specified directory. Additionally a comma-separated value (CSV) file is updated at each new stage location with the spatial coordinates of the stage. Finally, upon the successful conclusion of the program, a CSV file containing all parameters is written to the save folder. Additionally, a composite array of each region is constructed, by tiling together maximum projections of each tiff stack.

**ANALYSIS**

Further analysis scripts take the output of the image scanning stage and segment cells (using CellProfiler binaries modularly; [cellprofiler.org](http://cellprofiler.org/)), extract features of interest, detect smFISH spots, and output a nested MATLAB data structure or CSV file containing cellular properties linked with colony properties. An additional script outputs the files to a format that may be used with FCS Express ([denovosoftware.com](http://www.denovosoftware.com/)).

Alternatively, the analysis files may be used to process arrays of images captured independently of the imaging stage using off-the-shelf software, or other imaging platforms entirely, such as Laser Scanning Cytometry if the files are named appropriately.

Further instructions are available online (see “Availability” above).

**RESULTS AND DISCUSSION**

The program has been successfully used independently and without supervision by other researchers in our laboratory. The program was demonstrated to accurately scan regions selected in the prescan. This is illustrated in Figures S8a, S8b, and S10, a comparison between the selected region from the prescan and the resulting high-resolution image.

**FIGURES**


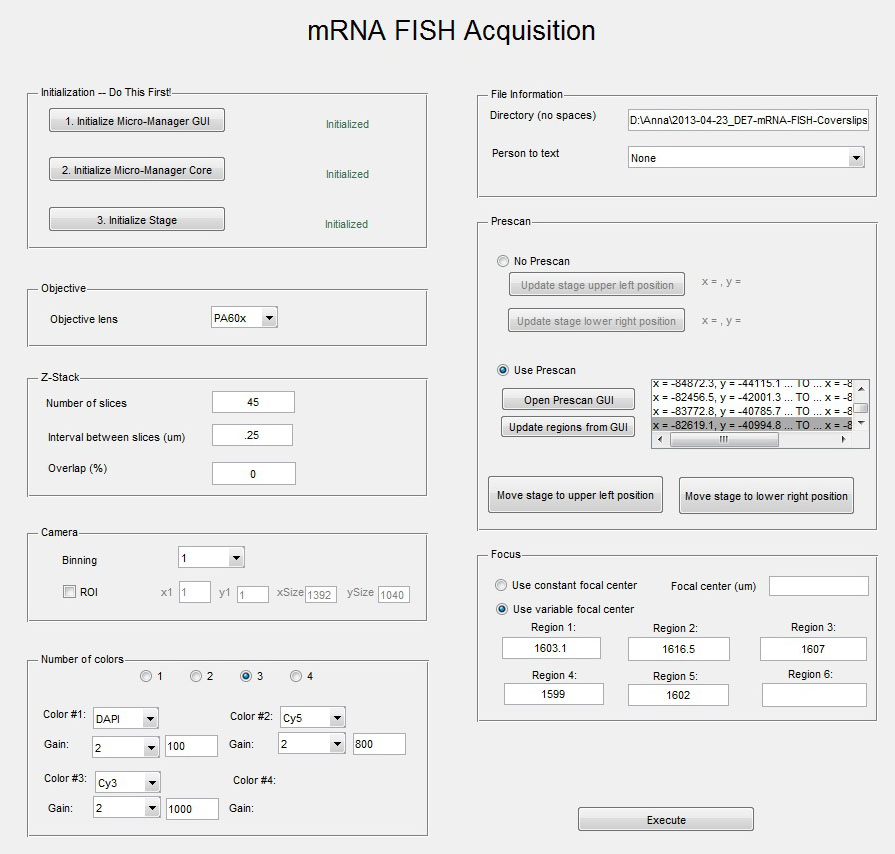


Figure S6. The main window of the mRNA FISH acquisition GUI.


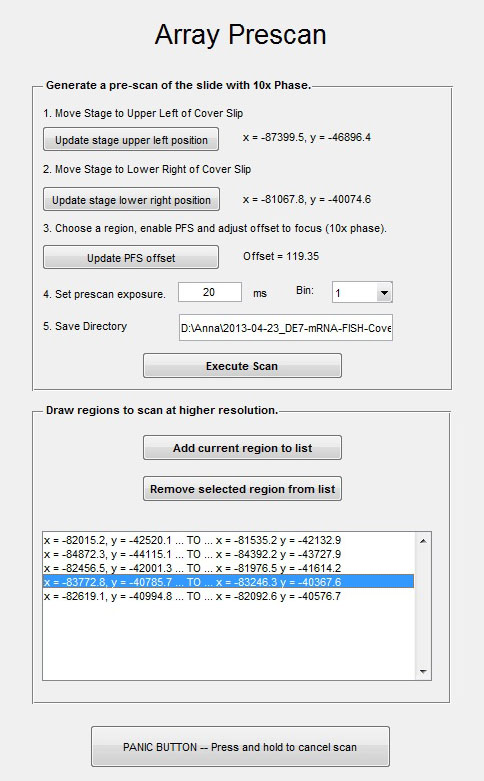


Figure S7. The prescan daughter window.


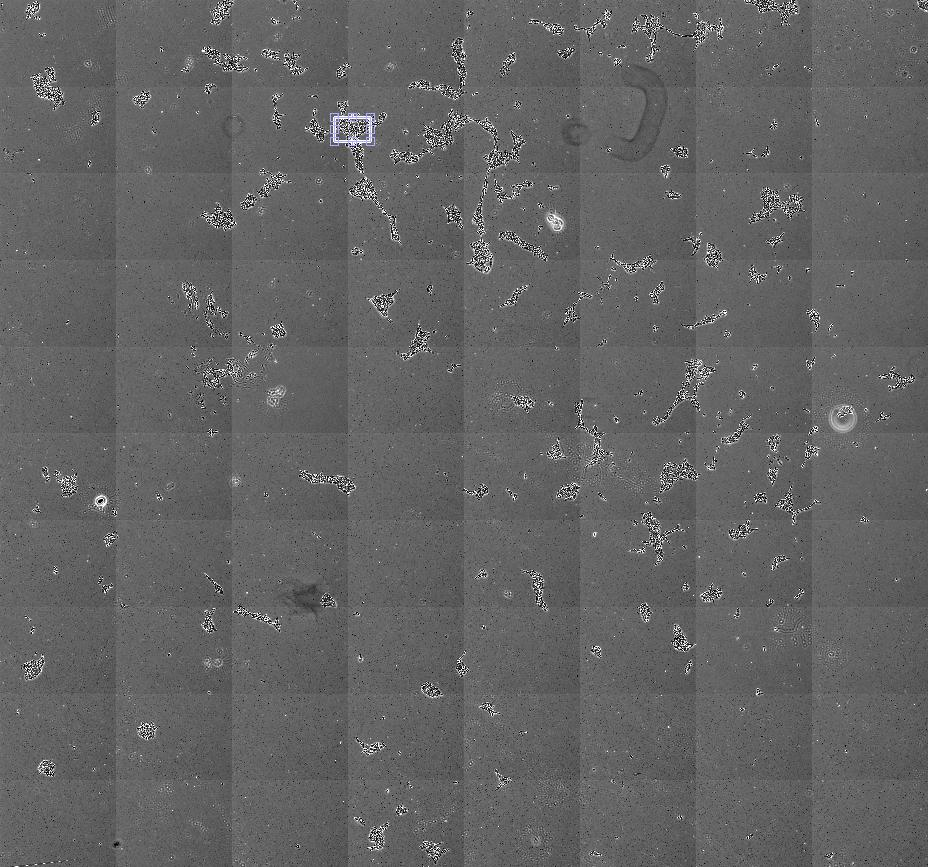


Figure S8a. Selection of regions for high-magnification imaging based on a low-magnification phase contrast prescan. This hESC culture was fixed for smFISH 48 hours after single-cell passaging onto matrigel, showing obvious heterogeneity at colony size, distribution etc.


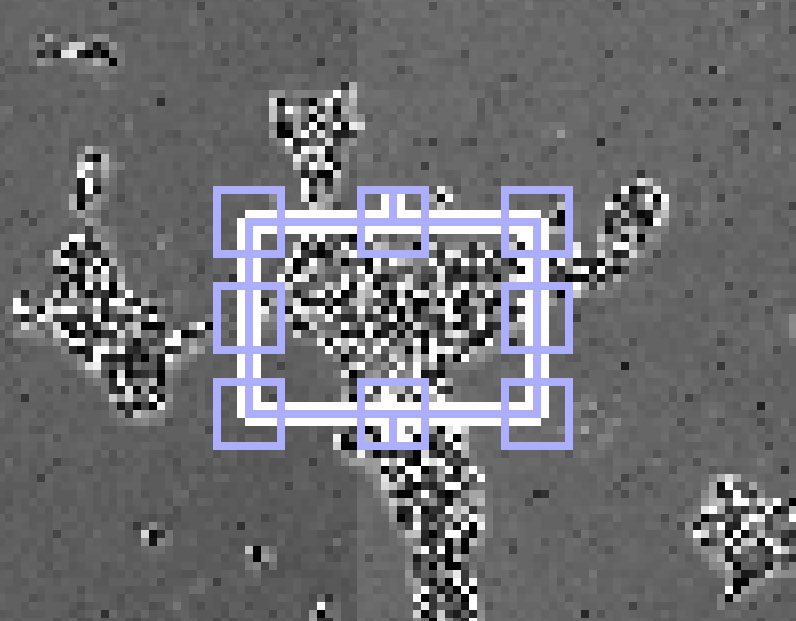


Figure S8b. Zoomed-in view of the region selected in Figure S8a.


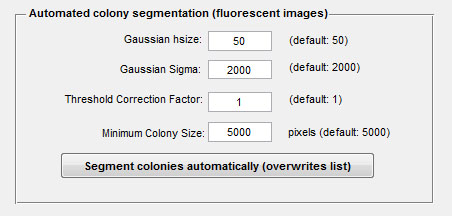


Figure S9. Additional options available with fluorescent pre-scans to automatically segment colonies and generate a list of colonies to image at high magnification. The colony list can then be flexibly reviewed and edited manually as needed.


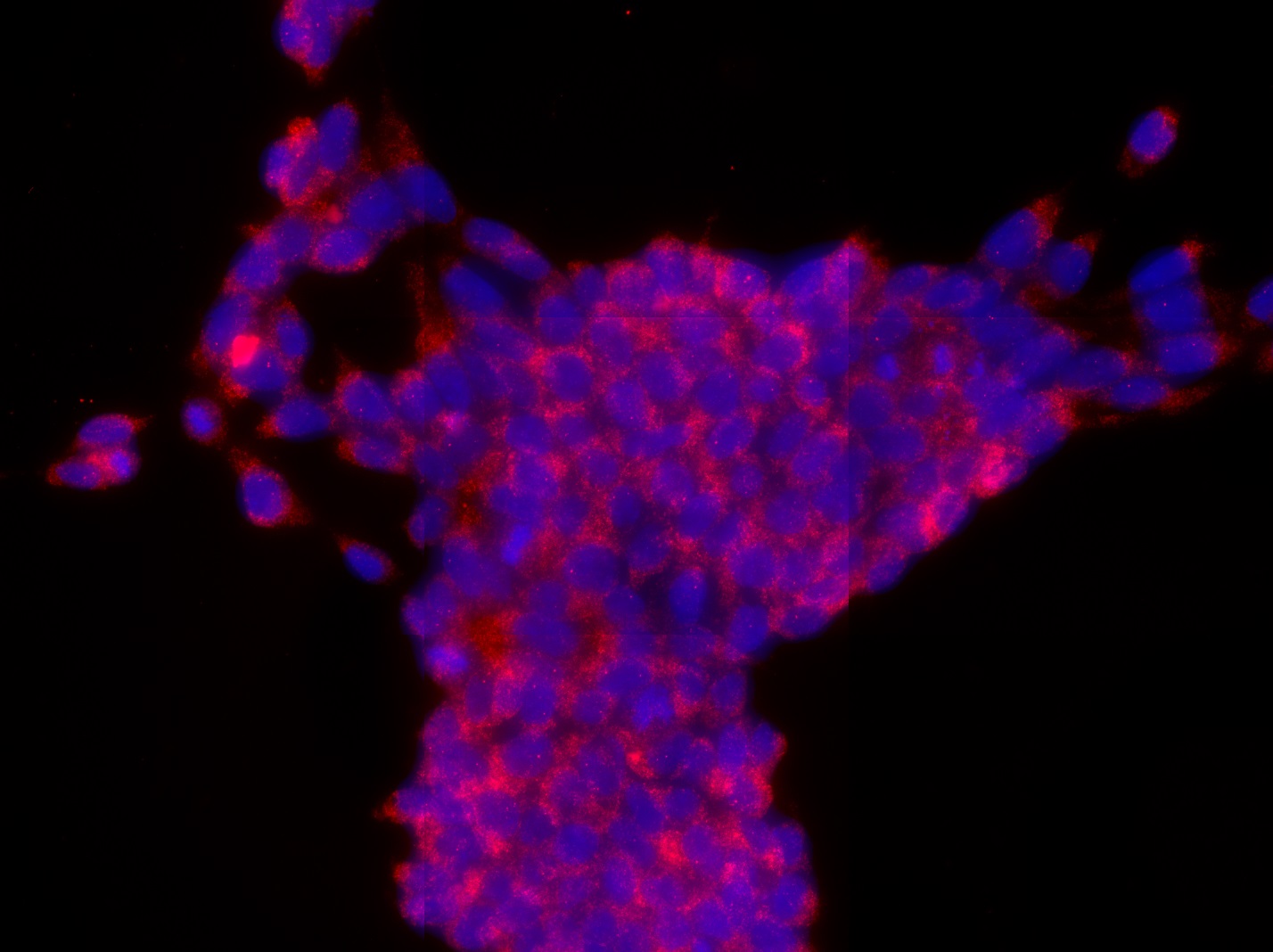


Figure S10. High-magnification imaging of region selected in Figures 3 and 4. Maximum projection images are automatically generated from z-stacks and titled into a regional array. The array is composed of 9 tiff stacks in a 3x3 grid. Red: smFISH of Oct4 transcripts, Blue: DAPI.
